# Intention to Pay for HPV Vaccination among Women of Childbearing Age in Vietnam

**DOI:** 10.3390/ijerph17093144

**Published:** 2020-04-30

**Authors:** Xuan Thi Thanh Le, Phuong Thi Ngoc Nguyen, Toan Thi Thanh Do, Thang Huu Nguyen, Huong Thi Le, Cuong Tat Nguyen, Giang Hai Ha, Chi Linh Hoang, Bach Xuan Tran, Carl A. Latkin, Roger C.M. Ho, Cyrus S.H. Ho

**Affiliations:** 1 Institute for Preventive Medicine and Public Health, Hanoi Medical University, Hanoi 100000, Vietnam; lethithanhxuan@hmu.edu.vn (X.T.T.L.); nguyenngocphuong2905@gmail.com (P.T.N.N.); dothithanhtoan@hmu.edu.vn (T.T.T.D.); nguyenhuuthang@hmu.edu.vn (T.H.N.); lethihuong@hmu.edu.vn (H.T.L.); bach.ipmph@gmail.com (B.X.T.); 2 Institute for Global Health Innovations, Duy Tan University, Da Nang 550000, Vietnam; nguyentatcuong@duytan.edu.vn; 3 Faculty of Medicine, Duy Tan University, Da Nang 550000, Vietnam; 4 Faculty of Pharmacy, Duy Tan University, Da Nang 550000, Vietnam; 5 Center of Excellence in Behavioral Medicine, Nguyen Tat Thanh University, Ho Chi Minh City 700000, Vietnam; chi.coentt@gmail.com (C.L.H.); pcmrhcm@nus.edu.sg (R.C.M.H.); 6 Bloomberg School of Public Health, Johns Hopkins University, Baltimore, MD 21205, USA; carl.latkin@jhu.edu; 7 Department of Psychological Medicine, Yong Loo Lin School of Medicine, National University of Singapore, Singapore 119228, Singapore; 8 Institute for Health Innovation and Technology (iHealthtech), National University of Singapore, Singapore 119077, Singapore; 9 Department of Psychological Medicine, National University Hospital, Singapore 119074, Singapore; cyrushosh@gmail.com

**Keywords:** HPV vaccination, cervical cancer, intention, women, Vietnam

## Abstract

The intention to pay for human papillomavirus (HPV) vaccination among women of childbearing age in Vietnam, where cervical cancer remains a significant public health concern, has been mostly lacking. To examine this issue, we conducted a cross-sectional study of 807 pregnant women in an urban and a rural district (Dong Da and Ba Vi) of Hanoi, Vietnam. The vast percentage of our respondents expressed a firm intention to vaccinate, especially women in rural areas (over 90.0%). However, on being informed of the current price of the HPV vaccine, their intention to vaccinate dropped to about one-fifth of overall respondents, i.e., only 4.4% of women in rural areas. It was also observed that the initial intention to get the HPV vaccination among women in the rural district was about ten times higher than that of women living in the metropolitan district. Those participants who had greater knowledge of cervical cancer and HPV vaccinations also had a significantly higher intention to vaccinate. Our findings underscore the need to develop a well-designed vaccination program in Vietnam and other countries in a similar situation to increase the adoption of HPV vaccination.

## 1. Introduction

Cervical cancer is the fourth most common cancer among women worldwide, and the second in developing regions [1]. It remains an enormous global health burden, affecting over 530,000 women and accounting for 270,000 deaths annually. Nearly 84% of cervical cancer patients and 87% of related deaths occur in developing parts of the world [1,2].

The launch of the human papillomavirus (HPV) vaccination globally in 2006–2007 was a life-saving intervention with the potential to prevent several common and high-risk types of HPV associated with cervical cancer [3]. So far, millions of women have been vaccinated. However, the global uptake of the HPV vaccination has been highly variable, especially in developing parts of the world. Low rates of HPV vaccination were found in Greece [4,5], Hong Kong [6], and Cambodia [7], though most women surveyed in these countries stated a strong intention to vaccinate [8,9]. In Southeast Asia, two-thirds of the women surveyed expressed a strong intention to get the HPV vaccine [7,10] while actual vaccination rates remained low.

To explain the enormous gap between intention and reality, earlier studies highlighted the cost of the vaccine as one of the chief obstacles to the more widespread utilization of the HPV vaccine [7,9]. In most developed parts of the world, where HPV vaccination is integrated into existing immunization programs, it is free of charge and not an economic burden for citizens. In less-developed regions, women’s intention to vaccinate dropped as soon as they learned they had to pay out of pocket or if the vaccination was considered a deductible [7]. Some studies conducted in Southeast Asia found that when the vaccination was free or priced exceedingly low (around $5), the rate of vaccination was extremely high [11,12,13,14].

Cervical cancer is the second-most-common cancer in women aged 15 to 44 in Vietnam [15]. In 2008, Vietnam conducted the HPV vaccination pilot program that utilized both a school- and community-based approach to increase the rate of vaccinations among children. This two-year pilot program sponsored by the Global Alliance for Vaccines and Immunization targeted 12-year-old girls. Although the pilot achieved a coverage rate of over 96.0%, the HPV vaccine is not included in the national immunization program currently. Compared with the global average cost of about $400 for a three-shot series of the HPV vaccine, the price of around $150–$195 in Vietnam is exceedingly low. However, this remains out of reach for most Vietnamese citizens, at nearly one-tenth of the average per capita income (about $2170) in 2016. Obstacles to the uptake of the HPV vaccination include price, parents who do not want to spend lots of money on their daughter (gender inequality), and inadequate knowledge, specifically among parents of the target population and especially in lower-middle-income countries such as Vietnam [16,17].

In this study of the Dong Da and Ba Vi districts of Hanoi, Vietnam, we assessed the intention to pay for HPV vaccination among pregnant women or those who recently gave birth. This is the first study to examine the intention to say yes to HPV vaccination among this specific group, which is important since females of reproductive age often play the role of the principal decision-makers in their families’ health care. Moreover, during this particular period, women also have more interest in vaccination programs—understanding their thought process is critical to the design and implementation of successful vaccination programs to increase the acceptance of the HPV vaccination in society and minimize the burden of both HPV and related diseases.

## 2. Materials and Methods

### 2.1. Study Setting, Sample Size, and Sampling Method

A cross-sectional study was conducted from February to June 2016 in two randomly chosen districts, Dong Da (urban) and Ba Vi (rural). These districts were purposely selected since they had experienced an Influenza A or Rubella epidemic in the last five years, had a close consultation with the Hanoi Provincial Health Department and expressed their commitment. Two health communes were selected as study locations based on the following conditions: they (1) had experienced an Influenza A or Rubella epidemic in the past five years; (2) were committed to participating in the study, and (3) had local vaccination policies and vaccines available at each site. These health communes could have more concerns about infectious diseases that could be prevented by our interest vaccination. Finally, a list of all women who were pregnant or who had given birth in the 12 months preceding the survey was collected from the relevant local agencies.

Since this project covered not only the HPV vaccine but also other vaccines (influenza, hepatitis B, tetanus, and rubella), we chose a study group for whom all these vaccines were relevant—women who were either pregnant or had recently given birth. Eligible participants in the survey were then randomly chosen by the health staff responsible for each cluster in a communal unit.

For sample size calculations, the level of significance was set to 0.05 and the power to 95%, and a two-sided test was utilized. Since there is no preexisting study of the coverage of HPV and other vaccines (influenza, hepatitis B, tetanus, or rubella) among reproductive-aged women in Vietnam, we set up *p* = 0.5 to represent the maximum variability of the population [18]. The sample was increased by 10% in case someone refused to participate or was absent during the collection period. The final sample size was thus about 400 women in each district and 807 in total. Participants were then selected by using a simple random sampling method in two communal health units in Dong Da and Ba Vi from a list of 975 and 470 eligible women, respectively. The interview response rate was > 95% for both districts.

Participants in a pre-evaluation study were adult women of reproductive age. They were enlisted by village health workers and the community health center staff responsible for managing vaccinations in their communities. These recruited women had to meet the following criteria to be included in the study: (1) aged 18–49 years at the time of the survey; (2) either pregnant or caring for a child under 12 months of age; and (3) currently living in the selected villages for at least one year and present in those villages at the time of the survey.

### 2.2. Measures and Instruments

A structured and face-to-face questionnaire in Vietnamese was used to obtain information on participants’ age, education level, current primary occupation, average monthly per capita income, and the current number of children. This questionnaire was developed based on the Vietnamese Ministry of Health’s guidelines for preventing infectious diseases and relevant references from the National Institute of Hygiene and Epidemiology [19]. The questionnaire was administered to five pregnant women with children under one year of age in Hanoi, Vietnam, before its utilization in this study. Of note, each item for the measure had alpha reliability greater than 0.6.

In this study, the intention to pay for the HPV vaccine was defined as the intention among unvaccinated women to receive the HPV vaccine after knowing its price. Sociodemographic characteristics measures include age (≤25/26−30/31+), marital status (with or without a spouse), child-birth status (having infants or pregnant), status of residence (permanent or no/temporary), educational level (high school or less and college or higher), occupation (blue-collar/white-collar/others), monthly household income categorized by the government with the 1,300,000 Vietnam Dong (VND) cut-off point (moderate or poor/near-poor), public health insurance card (yes/no) and self-reported health status (good vs. neutral/not good). Seven detailed questions on cervical cancer or five items on HPV vaccinations were asked of those who had knowledge about either. The detailed findings of these questions are shown in Table S1. One point was given for each correct answer, and the points were summed up to create a summary knowledge score. A high knowledge score means that a participant is relatively knowledgeable about cervical cancer or HPV vaccination. Each summary knowledge score was then divided into two groups: good and not good [20]. Women were categorized in the “good knowledge” group if their score was above the mean, and in the “not good knowledge” group if it was less than the mean. Besides, attitude toward HPV vaccination was measured whether the participants self-assess the vaccination as necessary (positive) or neutral or unnecessary (neutral or negative).

### 2.3. Statistical Analysis

Descriptive analysis was used to assess all sociodemographic variables. Either Pearson’s chi-square test or Fisher’s exact test (where the expected count for any particular cell is less than 5) was then employed in the bivariate analysis of all categorical variables. A *p*-value of less than 0.05 was considered statistically significant. A logistic regression analysis was used to evaluate the association between the intention to pay for HPV vaccinations and sociodemographic, knowledge-related, and attitudinal factors as well; the odds ratios (ORs) and 95% confidence intervals (CIs) were calculated. The regression models were separately examined by residency status. All statistical analyses were performed using STATA software version 14 (Stata Corp. L.P., College Station, TX, USA).

### 2.4. Ethical Considerations

This study was approved by the Ethics Committee of Hanoi Medical University (Code number: 184/HMU-IRB dated 14 November 2015). Following a face-to-face explanation of the study given by trained healthcare workers at the Hanoi Medical University, all participants gave verbal consent prior to their participation in the study, acknowledging their full understanding of the study’s purpose, their rights to withdraw from the study at any time, and the protection and confidentiality of the collected data.

## 3. Results

Table 1 shows the distribution of significant characteristics among participants. A total of 807 participants participated in the study, two-thirds of whom were younger than 30 years. More young women from the rural district (Ba Vi) took part in the survey than from the urban one (Dong Da). Most participants from non-metropolitan areas had lower education levels and were blue-collar workers or peasants. As a result, most of the rural participants were reported to be living in poverty. The coverage of health insurance, therefore, was higher among urban participants, even though there was no difference in health status.

**Figure 1:**
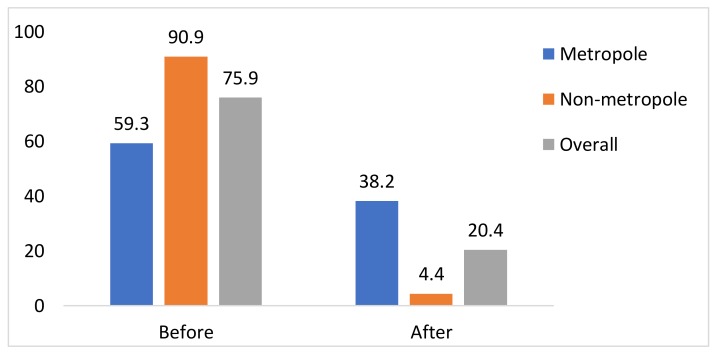
Intention to receive the HPV vaccination before/after knowing the price (*n* = 769).

Figure 1 shows the proportion of nonvaccinated women willing to receive the HPV vaccine before and after knowing its price. A considerable percentage of respondents expressed a firm intention to vaccinate, especially in rural areas (over 90.0%), however, after being informed of the current price of the HPV vaccination, their intentions dropped swiftly to about one-fifth of all respondents and to 4.4% for women in rural areas.

Table 2 shows participants‘ intention to get an HPV vaccination before learning its price. Rural females, and even those who lacked knowledge of the benefits of HPV vaccinations, were the most likely to want to get vaccinated. Older women and those with infants, permanent residence, and higher household income had relatively strong intentions to vaccinate. This intention was also seen among women with good knowledge of cervical cancer and a positive attitude toward vaccines. The association between the intention to receive HPV vaccination and monthly household income was significant among participants with both permanent and temporary residency status.

Table 3 demonstrates factors associated with the intention to receive the HPV vaccine (that is, before receiving price information) for unvaccinated women in each subgroup. Overall, women who lived in the countryside had lower educational attainment, were blue-collar workers/peasants, and were not covered by health insurance. Of these, those who were temporary residents and relatively older were less likely to want to get vaccinated.

Among the metropolitan group, those who had adequate knowledge of cervical cancer and HPV vaccination, as well as a positive attitude toward vaccinations, were more likely to want to get vaccinated.

In the non-metropolitan group, those who had greater knowledge of and a positive attitude toward HPV vaccination were more likely to want to get vaccinated. However, the more impoverished the household, the weaker the intention to vaccinate.

The multivariable logistics for the intention to receive the HPV vaccine (before knowing its price) among nonvaccinated women are displayed in Table 4. A strong correlation was noted between location and intention to vaccinate against HPV. Women from poor or near-poor households, especially those in rural areas, were the least likely to want to be vaccinated against HPV. The strongest intention was seen among those with a good knowledge of cervical cancer and HPV vaccinations and a positive attitude.

## 4. Discussion

This is the first study to quantitatively investigate the intention to pay for HPV vaccination among women of childbearing age in Vietnam, after controlling for residency status. Cervical cancer remains a significant public health concern in this country. As others have noted, we found that the overwhelming majority of our respondents expressed a strong intention to accept the HPV vaccination [8,9,21,22]. However, a drop in this intention was observed after the women learned its price, similar to previous results [8,12,23,24,25]. Additionally, none of the participants categorized as being from a poor/near-poor household was vaccinated against HPV. It was correspondingly predicted in a PATH study in 2009 that cost could be one of the chief obstacles to the uptake of the HPV vaccine in Vietnam [26]. Diverse studies conducted in developing parts of the world have highlighted high cost as an obstacle to vaccine acceptance [27,28], indicating that economic constraints keep women from taking advantage of this important health service even where it is available. Strategies to promote the HPV vaccine will need to pay particular attention to some key demographic trends. Women with temporary residency status, with relatively low levels of educational attainment, and who come from poor households are the least likely to want to accept the HPV vaccine.

In our study, the intention to accept HPV vaccination was about ten times higher among rural women (Figure 1) than among urban women after knowing the price, corroborating findings from populations in 2016 [29]. This could be because the rural participants did not take the price into account. Our data also clearly demonstrated the difference in their intention before and after knowing the price. The intention to vaccinate dropped quickly from more than almost all rural women to less than 5% before and after knowing the price. In Vietnam, the vaccine against HPV was promoted using the name “cervical cancer vaccine” to emphasize the goal of cervical cancer prevention [26]. In prior studies, rural women were found to have a higher cervical cancer rate, so they might correspondingly have had greater concern about cervical cancer and are more interested in its prevention [29,30,31].

Our study received strong support from local health workers in each collection unit. The relationship between physicians and communities may be more robust in the countryside in comparison with urban zones, as cited in a recent US study [29]. Therefore, rural women, who are more likely to trust their health workers could agree to this vaccination more easily, even though nearly half of them had not heard of it before participating in our survey.

As foreseen, the results clearly show that those with preexisting knowledge of cervical cancer and HPV vaccinations also had significantly greater intention to get vaccinated. This is in line with another study observing that people’s level of knowledge appears to significantly influence their intention to vaccinate [4,5,6,32,33,34,35]. The “health belief model” has been utilized widely to justify several predictors of potential vaccine uptake [36]. According to this model, women with less knowledge of cervical cancer and HPV vaccinations are believed to have a lower perception of the risks and severity of an HPV infection and of the benefits of the HPV vaccine, which may lead to their lower intention of getting vaccinated against HPV [36]. Further, as a decade has gone by since the approval of the HPV vaccine, it might be assumed that information about it has been spread widely during this period and has generated a more positive attitude among women toward the vaccine. Thus, knowledge of cervical cancer/HPV vaccinations is changing behavior and increasing the number of women getting vaccinated [35,37].

Some existing limitations of the present study may affect the interpretation of our findings. First, data were employed in a cross-sectional study in the pre-evaluation in 2016, so it is beyond the scope of the present study to determine any causal relationship. Second, participants in this study (women of childbearing age) were mainly out of the recommended age for HPV vaccination (9–26 years old). Thus, their intention to vaccinate themselves could be quite low compared to their intention to vaccinate their daughters. To deal with this limitation, we recommend that their intention to vaccinate their daughters against HPV be queried in the post-evaluation phase. Lastly, this study only focused on women of pregnancy or child-birth age; thus, our result may not be representative of the entire population of women.

Despite these limitations, our study has several strengths. It is the first study to investigate the association between several factors and the intention to get vaccinated against HPV among women of childbearing age in Vietnam. The findings could call for public health policy to implement a step-by-step strategy for cervical cancer prevention. The study sample of women of childbearing age, a child’s primary caretaker and health keeper for each family is also a strength. As others have stated, decisions regarding vaccines, including against HPV, are primarily made by the mother; other family members play either a small role or no role at all [16,17]. Mothers’ intentions to vaccinate themselves and their daughters against HPV could be sharply limited due to a lack of knowledge about cervical cancer and HPV vaccinations. Therefore, the most important thing to do is to understand and improve parental knowledge about HPV vaccination and tackle obstacles to its future uptake.

Collectively, based on the present findings, education programs would do well to build on existing knowledge, focusing on women (and particularly mothers), while simultaneously addressing common misconceptions. Since knowledge is a necessary precursor for health-protective behaviors, including the decision to get vaccinated, addressing knowledge gaps may enable people to make better choices. Further, education campaigns (including the vaccine’s promotion via leaflets) should take advantage of community health centers, where rural women go to get general vaccinations. Also, to deal with the high cost of this expensive vaccine, a two-dose schedule should be carefully considered, and the cost reduced to at least one-third of the current price to make it affordable. Further, integration of this vaccination into our national immunization program should be taken into the government’s consideration since it could be a life-saving vaccine for the public. The two-dose schedule and public tenders could lead to important price rebate opportunities.

Looking ahead, a qualitative study is needed to better understand women’s outlook on the HPV vaccine and the particular obstacles to vaccination in Vietnam.

## 5. Conclusions

An overwhelming majority of study respondents (women of childbearing age) expressed a strong intention to get vaccinated against HPV across both urban and rural areas of Hanoi, the capital of Vietnam. However, a quick drop in the intention to vaccinate was observed once these same women were informed of the vaccine price. Our findings thus underscore the need to develop a well-designed vaccination program in Vietnam and other countries in a similar situation to increase the uptake of HPV vaccination.

## Figures and Tables

**Table 1:** Participant’s demographic characteristics.

Variables	Overall		Urban		Rural		*p*-Value ^1^
	*n*	%	*n*	%	*n*	%	
Total	807	100.0	400	49.6	407	50.4	
**Age group**							
≤25 years old	222	27.5	64	16.0	158	38.8	<0.001
26–30 years old	302	37.4	156	39.0	146	35.9	
>30 years old	283	35.1	180	45.0	103	25.3	
**Marital status**							
With spouse	801	99.3	398	99.5	403	99.0	0.43 ^2^
Without spouse	6	0.7	2	0.5	4	1.0	
**Child-birth status**							
Having infants (0–1 years)	609	75.5	299	74.8	310	76.2	0.64
Pregnant	198	24.5	101	25.2	97	23.8	
**Residential status**							
Permanent residence	706	87.5	320	80.0	386	94.8	<0.001
Temporary residence	101	12.5	80	20.0	21	5.2	
**Educational attainment**							
College or higher	424	55.5	322	80.5	102	25.1	<0.001
High school or lower	383	47.5	78	19.5	305	74.9	
**Occupation ^3^**							
White collar	252	31.2	213	53.3	39	9.6	<0.001
Blue collar/Peasants	284	35.2	16	4.0	268	65.9	
Others	271	33.6	171	42.7	100	24.5	
**Household monthly income per person ^4^**							
Moderate or higher	598	74.1	382	95.5	216	53.1	<0.001
Poor or near poor	209	25.9	18	4.5	191	46.9	
**Health insurance card**							
Yes	509	63.1	316	79.0	193	47.4	<0.001
No/No response	298	36.9	84	21.0	214	52.6	
**Self-reported health status**							
Good	502	62.2	255	63.8	247	60.7	<0.001
Central or not good	305	37.8	145	36.2	160	39.3	
**Vaccinated with HPV vaccination**							
Yes	38	4.7	36	9.0	2	0.5	<0.001 ^2^
No	769	95.3	364	91.0	405	99.5	

^1^*p*-value was obtained from Pearson’s chi-square test. ^2^*p*-value was obtained from Fisher’s exact test. ^3^ Blue-collar/peasants: workers in the sales and service sectors, craftspeople, skilled laborers, machine operators, peasants; white-collar: managers, professionals, experts, engineers, office workers; and others: students, unemployed, housewives. ^4^ Household income per person was categorized as moderate if the income was ≥ VND 1,300,000, or poor/near-poor for the rest.

**Table 2:** Demographic differences and the intention to vaccinate against HPV (%).

Variable	Overall (*n* = 769)	Metropolitan (*n* = 364)	Nonmetropolitan (*n* = 405)
Total	75.9	59.3	90.9
**Age**			
≤25 years old	83.3	66.1	89.8
26–30 years old	75.7	58	91.7
>30 years old	70.4	58.1	91.3
*p*-value	0.004	0.51	0.84
**Marriage status**			
With spouse	75.9	59.1	91
Without spouse	83.3	100	75
*p*-value	0.67	0.24	0.27
**Child-birth status**			
Having infants (0–1 years)	77.3	62.3	90.6
Pregnant	71.8	50.6	91.8
*p*-value	0.13	0.049	0.73
**Residential status**			
Permanent residence	77.2	59.5	90.6
No/Temporary residence	67	58.9	95.2
*p*-value	0.03	0.93	0.48
**Educational attainment**			
College or higher	68.7	60.6	92
High school or lower	83.3	54.6	90.5
*p*-value	<0.001	0.34	0.65
**Occupation ^1^**			
White collar	65.5	60.1	92.1
Blue collar/Peasants	87	43.8	89.6
Others	73	60	93.9
*p*-value	<0.001	0.43	0.42
**Household monthly income per person ^2^**			
Moderate or higher	73.4	59.3	96.3
Poor or near poor	82.8	61.1	84.8
*p*-value	0.007	0.88	<0.001
**Health insurance card**			
Yes	73.3	60.4	92.2
No/No response	80.3	55.6	89.7
*p*-value	0.03	0.43	0.38
**Self-reported health status**			
Good	75.5	59.7	90.2
Central or not good	76.7	58.8	91.9
*p*-value	0.7	0.88	0.57
**Knowledge of cervical cancer ^3^**			
Good	83.7	68.6	95.5
Not good	67.9	51	85.4
*p*-value	<0.001	0.001	<0.001
**Knowledge of HPV vaccinations**			
Good	81.1	71.8	92.4
Not good	72.3	47.6	90
*p*-value	0.005	<0.001	0.42
**Attitude toward HPV vaccinations**			
Positive	82.7	69.2	93.2
Neutral/negative	44.4	27.1	74
*p*-value	<0.001	<0.001	<0.001

^1^ Blue-collar/peasants: workers in the sales and service sectors, craftspeople, skilled laborers, machine operators, peasants; white-collar: managers, professionals, experts, engineers, office workers; and others: students, unemployed, housewives. ^2^ Household income per person was categorized as moderate if the income was ≥ VND 1,300,000, or poor/near-poor for the rest. ^3^ For those who had heard about cervical cancer/HPV vaccination only.

**Table 3:** Univariate logistic regression for intention to receive the HPV vaccination.

Variables	Overall		Metropolitan		Nonmetropolitan	
	OR ^1^	95% CI ^2^	OR ^1^	95% CI ^2^	OR ^1^	95% CI ^2^
**Location** (Metropolitan = ref ^3^)	6.81	4.58–10.14				
**Age** (≤25 years old = ref)						
26–30 years old	0.62	0.40–0.98	0.71	0.37–1.35	1.26	0.57–2.76
>30 years old	0.48	0.31–0.74	0.71	0.38–1.32	1.19	0.50–2.79
**Residency status** (Permanent residence = ref)	0.60	0.38–0.96	0.98	0.58–1.65	2.07	0.27–15.87
**Educational attainment** (College or higher = ref)	2.26	1.60–3.19	0.78	0.47–1.29	0.83	0.37–1.87
**Household monthly income** (Moderate or higher = ref)	1.74	1.16–2.61	1.08	0.41–2.86	0.22	0.10–0.49
**Health insurance card** (No = ref)	0.67	0.47–0.96	1.22	0.74–2.01	1.36	0.68–2.70
**Self-reported health status** (No good = ref)	0.94	0.66–1.32	1.03	0.67–1.59	0.81	0.40–1.65
**Knowledge of cervical cancer** (Not good = ref)	2.42	1.71–3.42	2.10	1.37–3.22	3.59	1.69–7.63
**Knowledge of HPV vaccinations** (Not good = ref)	1.64	1.16–2.32	2.80	1.81–4.32	1.35	0.65–2.83
**Attitude toward HPV vaccinations** (Neutral or negative = ref)	5.95	4.00–8.86	6.05	3.52–10.40	4.85	2.28–10.32

^1^ OR: odds ratio; ^2^ CI = confidence interval; ^3^ ref = reference.

**Table 4:** Multivariable logistics for the intention to receive the HPV vaccine ^1^.

Variables	Overall		Metropolitan Group		Nonmetropolitan Group	
	aOR ^2^	95% CI ^3^	aOR ^2^	95% CI ^3^	aOR ^2^	95% CI ^3^
**Location** (Metropolitan = ref ^4^)	10.53	5.58–19.86				
**Age** (≤25 years old = ref)						
26–30 years old	0.75	0.44–1.29	0.47	0.23–0.99	1.50	0.64–3.54
>30 years old	0.79	0.47–1.35	0.57	0.28–1.15	1.35	0.53–3.48
**Residency status** (Permanent residence = ref)	1.37	0.78–2.38	1.37	0.75–2.51	3.72	0.40–34.44
**Educational attainment** (College or higher = ref)	1.03	0.62–1.71	0.81	0.44–1.49	1.68	0.65–4.33
**Household monthly income** (Moderate or higher = ref)	0.48	0.26–0.90	1.48	0.48–4.58	0.21	0.09–0.52
**Health insurance card** (No = ref)	1.08	0.68–1.71	1.09	0.60–1.97	1.05	0.49–2.25
**Self-reported health status** (No good = ref)	0.84	0.56–1.26	0.96	0.59–1.56	0.62	0.29–1.32
**Knowledge of cervical cancer** (Not good = ref)	1.72	1.14–2.61	1.44	0.88–2.36	2.46	1.08–5.63
**Knowledge of HPV vaccinations** (Not good = ref)	1.60	1.06–2.42	1.89	1.17–3.04	0.89	0.39–2.02
**Attitude toward HPV vaccinations** (Neutral or negative = ref)	4.37	2.73–6.98	5.25	2.89–9.52	3.62	1.51–8.66

^1^ Adjusted for all variables in the model. ^2^ aOR = adjusted odds ratio. ^3^ CI = confidence interval; ^4^ ref = reference.

## Supplementary Materials

The following are available online at https://www.mdpi.com/1660-4601/17/9/3144/s1, Table S1: Knowledge of cervical cancer and HPV vaccine questions.
