# Supplementary material for: Intention to Pay for HPV Vaccination among Women of Childbearing Age in Vietnam
Source: Int J Environ Res Public Health. 2020 Apr 30;17(9):3144. doi: 10.3390/ijerph17093144 (PMC7246945; doi:10.3390/ijerph17093144)
Supplement: Supplementary file 1 [file ijerph-17-03144-s001.pdf]

Table S1. Knowledge of cervical cancer and HPV vaccine questions.

Question	True	False	Unknown/ No Response
**Cervical cancer knowledge**	Total score: 7		
Cervical cancer is an inherited disease	0	1	0
Cervical cancer is caused by a virus	1	0	0
Cervical cancer is an infectious disease	0	1	0
**Population at high cervical cancer risk**			
Women who have given birth	1 point if pointing to at least an answer		
Elderly people			
People with multiple sexual partners			
Women with poor (gynecological) hygiene behaviors			
**Signs of cervical cancer**			
Abnormal vaginal bleeding	1 point if pointing to at least an answer		
Unusual discharge from the vagina			
Urinary incontinence			
Bleeding after intercourse			
Pain during sexual intercourse			
Back pain			
Cervical cancer can be prevented	0	1	0
HPV vaccine is one of the most effective ways to prevent cervical cancer	0	1	0
**Knowledge of HPV vaccine**	Total score: 5		
HPV vaccines include 3 shots in Vietnam	1	0	0
HPV vaccines should be taken at ages 9–26 years	1	0	0
Women who do not engage in sex can still get vaccinated	1	0	0
Sexually active women should get an HPV test before being vaccinated	1	0	0
**Side effect of HPV vaccines**			
Redness or swelling	1 point if pointing to at least an answer		
Headache			
Fever (mild or moderate)			
